# Editorial: Safe and Trustworthy Machine Learning

**DOI:** 10.3389/fdata.2021.731605

**Published:** 2021-08-06

**Authors:** Bhavya Kailkhura, Pin-Yu Chen, Xue Lin, Bo Li

**Affiliations:** ^1^ Lawrence Livermore National Laboratory, Livermore, CA, United States; ^2^ IBM Research, Yorktown Heights, NY, United States; ^3^ Electrical and Computer Engineering, Northeastern University, Boston, MA, United States; ^4^ Computer Science, University of Illinois Urbana-Champaign, Champaign, IL, United States

**Keywords:** robust machine learning, safe machine learning, trust, deep neural network, adversarial example defense

Machine learning (ML) provides incredible opportunities to answer some of the most important and difficult questions in a wide range of applications. However, ML systems often face a major challenge when applied in the real world: the conditions under which the system was deployed can differ from those under which it was developed. Recent examples have shown that ML methods are highly susceptible to minor changes in image orientation, minute amounts of adversarial corruptions, or bias in the data. Susceptibility of ML methods to test-time shift is a major hurdle in a universal acceptance of these solutions in several high-regret applications. To overcome this challenge, in this research topic “Safe and Trustworthy Machine Learning”, a wide range of solutions are contributed as potentially viable solutions to address trust, safety and security issues faced by ML methods.

## Papers Included in This Research Topic

Song, et al., considered the problem of dataset shift detection for safety-critical graph applications. The authors proposed a practical two-sample test approach for shift detection in large-scale graph structured data.

Anirudh, et al., considered the problem of post-hoc interpretability tasks, such as, prediction explanation, noisy label detection, adversarial example detection. The authors introduced MARGIN, a simple yet general approach, that exploits ideas rooted in graph signal analysis to determine the most influential nodes in a graph to solve the aforementioned tasks.

Majumdar, et al., considered the problem of mitigation of bias arising due to unbalanced representation of sub-groups in the training data. The authors proposed a bias mitigation algorithm to generate Subgroup Invariant Perturbation (SIP) which when added the input dataset reduces the bias in model predictions.

Huang, et al., showed that seq2seq models, successful in natural language correction, is also applicable in programming language correction. Their results show that seq2seq models can provide suggestions to potential errors and have a decent correct rate in code auto-correction task.

Qayyum, et al., conducted a systematic evaluation of literature of cloud-hosted ML/DL models along both the important dimensions -- attacks and defenses -- related to their security. The authors identified the limitations and pitfalls of the analyzed papers and highlight open research issues that require further investigation.

Berghoff, et al., presented a comprehensive list of threats and possible mitigations of IT security of connectionist artificial intelligence (AI) applications. AI-specific vulnerabilities such as adversarial attacks and poisoning attacks as well as their AI-specific root causes are discussed in detail. The article concluded that single protective measures are not sufficient but rather multiple measures on different levels must be combined to achieve a minimum level of IT security for AI applications.

Kusters, et al., analyzed key challenges to interdisciplinary AI research, and deliver three broad conclusions: 1) future development of AI should not only impact other scientific domains but should also take inspiration and benefit from other fields of science, 2) AI research must be accompanied by decision explainability, dataset bias transparency as well as development of evaluation methodologies and creation of regulatory agencies to ensure responsibility, and 3) AI education should receive more attention, efforts and innovation from the educational and scientific communities.

## Conclusions and Outlook

The papers included in this research topic “Safe and Trustworthy Machine Learning” discussed some promising solutions, highlighted open research issues, and offered visionary perspectives regarding trust, safety and security issues faced by machine learning. We hope that challenges and potential solutions presented here will help researchers better understand the current limitations of machine learning methods and motivate future work in the direction of developing trustworthy, safe, and robust machine learning methods, and their applications to high-regret application areas.

